# Quality of life in a real-world cohort of advanced breast cancer patients: a study of the SONABRE Registry

**DOI:** 10.1007/s11136-020-02604-4

**Published:** 2020-08-20

**Authors:** Anouk K. M. Claessens, Bram L. T. Ramaekers, Dorien J. A. Lobbezoo, Roel J. W. van Kampen, Maaike de Boer, Agnes J. van de Wouw, M. Wouter Dercksen, Sandra M. E. Geurts, Manuela A. Joore, Vivianne C. G. Tjan-Heijnen

**Affiliations:** 1 Department of Medical Oncology, GROW-School for Oncology and Developmental Biology, Maastricht University Medical Center, PO BOX 5800, 6202 AZ Maastricht, The Netherlands; 2 Department of Medical Oncology, Zuyderland Medical Center, PO BOX 5500, 6130 MB Sittard-Geleen, The Netherlands; 3 Department of Clinical Epidemiology and Medical Technology Assessment (KEMTA), Maastricht University Medical Centre, PO BOX 5800, 6202 AZ Maastricht, The Netherlands; 4 Department of Internal Medicine, VieCuri Medical Center, PO BOX 1926, 5900 BX Venlo, The Netherlands; 5 Department of Internal Medicine, Máxima Medical Center, PO BOX 90052, 5600 PD Eindhoven, The Netherlands

**Keywords:** Advanced breast cancer, Quality of life, EQ-5D, Real-world, Health utilities

## Abstract

**Purpose:**

We aimed to evaluate quality of life (QoL) using the European Quality of Life Five-Dimensions questionnaire (EQ-5D-3L) in a real-world cohort of Dutch advanced breast cancer (ABC) patients. Secondary, we reported differences in QoL between subgroups of patients based on age, comorbidity, tumor-, and treatment characteristics, and assessed the association of duration of metastatic disease and time to death with QoL.

**Methods:**

ABC patients who attended the outpatient clinic between October 2010 and May 2011 were asked to fill out the EQ-5D-3L questionnaire. Patient-, disease-, and treatment characteristics were obtained from the medical files. Health-utility scores were calculated. Subgroups were described and compared for utility scores by parametric and non-parametric methods.

**Results:**

A total of 92 patients were included with a median utility score of 0.691 (Interquartile range [IQR] 0.244). Patients ≥ 65 years had significantly worse median utility scores than younger patients; 0.638 versus 0.743, respectively (*p* = 0.017). Moreover, scores were significantly worse for patients with versus those without comorbidity (medians 0.620 versus 0.725, *p* = 0.005). Utility scores did not significantly differ between subgroups of tumor type, type of systemic treatment, number of previous palliative treatment(s), or number or location of metastatic site(s). The remaining survival was correlated with utility scores (correlation coefficient (*r*) = 0.260, *p* = 0.0252), especially in the subgroup < 65 years (*r* = 0.340, *p* = 0.0169), whereas there was no significant correlation with time since metastatic diagnosis (*r* = − 0.106, *p* = 0.3136).

**Conclusion:**

Within this real-world cross-sectional study, QoL was significantly associated with age, comorbidity, and remaining survival duration. The observation of a lower QoL in ABC patients, possibly indicating the last period of life, may assist clinical decision-making on timing of cessation of systemic antitumor therapy.

## Background

Advanced breast cancer (ABC), defined as metastatic breast cancer (stage IV), is a major cause of death among women worldwide [1]. Despite improving outcome of patients with ABC due to the introduction of new treatment agents and strategies, the disease remains largely incurable [2–4]. Thus, treatment focuses on both quality- and prolongation of life. Previous studies have shown that the quality of life (QoL) of these patients is positively associated with response to antitumor treatment, time to progression, and survival, but negatively associated with toxicity [5–8]. It is therefore essential to report on QoL in addition to clinical parameters to determine the health benefit of a new treatment.

There are different questionnaires suitable for evaluation of QoL among patients with ABC ranging from disease specific tools such as the European Organization for Research and Treatment for Cancer Quality of Life Questionnaire (EORTC QLQ-C30) to more generic instruments that allow comparison of QoL across diseases. Here we use the European Quality of Life Five-Dimensions questionnaire (EQ-5D) to describe QoL and estimate a single summary index value rated on a scale from 0 (dead) to 1 (full health) [9]. The EQ-5D is the most widely used generic instrument to obtain such an utility score that reflects the overall health-state of participants according to the preferences of the general population of a country or region. In several European countries, this standardized questionnaire is preferred as a key component in cost-utility analysis [10, 11], and recently it was highlighted to be the most commonly cited multi-attribute utility instrument [12]. It has been used in clinical trials as well as observational studies for different types and stages of treatment of malignant diseases [13].

Despite the increased awareness of evaluation of QoL in addition to clinical outcomes, QoL and health-utility are underreported and not uniformly addressed in most breast cancer studies [8, 14–17]. Gaining insights into the QoL and the factors influencing this outcome is especially important for advanced stages of breast cancer, as the goal for these patients does comprise not only prolonging life, but also optimizing the QoL, considering treatments given in this disease stage are of palliative intend. Understanding which factors influence the QoL of these patients promotes individualized high-quality care. The primary aim of our analysis was to evaluate QoL by means of the EQ-5D in patients with ABC in a real-world Dutch cohort. In addition, we reported differences in QoL between subgroups of patients based on age, comorbidity, tumor-, and treatment characteristics, and assessed the association of duration of metastatic disease and time to death with QoL.

## Methods

### Southeast Netherlands Advanced BREast cancer (SONABRE) Registry

This study was part of the SONABRE Registry (NCT03577197), an ongoing real-world multi-center cohort study of ABC patients. This registry aims to include all patients diagnosed with ABC between 2007 and 2021 in 12 hospitals in the Southeast of the Netherlands. The study reported here was approved by the Medical Research Ethics Committee of Maastricht University Medical Center as part of the SONABRE Registry. Informed consent was obtained from all patients prior to inclusion.

### Study population

Patients with ABC (stage IV disease) were identified from four hospitals participating in the SONABRE Registry, one academic and three teaching hospitals. Patients were eligible irrespective of being on active treatment or type of treatment. From October 2010 to May 2011, all ABC patients visiting the outpatient ward were invited by their treating oncologist to fill out the EQ-5D questionnaire, after general oral instruction during the informed consent procedure by their treating oncologist. They were invited only once, even if they attended the outpatient clinic more than once during the recruitment period.

### Data collection

We used the Dutch version of the EQ-5D-3L questionnaire [13], which was provided by the medical oncologist after obtaining informed consent, and was filled out immediately. This questionnaire comprises five dimensions describing QoL on mobility, self-care, usual activities, pain/discomfort, and anxiety/depression. Each dimension has three answering categories: no problems (level 1), some problems (level 2), and extreme problems (level 3) [13]. We used country-specific tariffs to calculate the overall health-state utility score from the reported items [18]. Additionally, patient, disease, and treatment characteristics were obtained from the medical files by oncologists with experience in the treatment of breast cancer patients. Death and if applicable, date of death, was evaluated for all included patients in December 2016.

### Study endpoints and statistical analyses

The primary study endpoint was to describe the health-utility score for the whole cohort. Previous studies on real-world QoL within breast cancer patients found several patient-, disease-, and treatment factors to exert a significant influence on the experienced QoL, including age [19, 20], comorbidities [20], type of therapy [19–24], number of lines of therapy [21, 24], location of metastases, [21, 22, 25], and disease progression [23, 26–28]. Therefor, our secondary endpoints included differences in QoL between subgroups of patients based on age (≥ 65 versus < 65 years), comorbidity according to the Charlson index (yes versus no and multiple subdivisions in comorbidity), tumor subtype (hormone receptor (HR) and human epidermal growth factor receptor-2 (HER2) status), current treatment type (endocrine, chemo- and/or targeted therapy), total number of prior palliative treatment lines, and number and metastatic site(s). As age has an influence on the experienced QoL also in the general population [29, 30], we reported the individual item scores of the EQ-5D-3L questionnaire for age groups ≥ 65 versus < 65 years. Additionally, we assessed the association of duration of metastatic disease and time to death with QoL.

The metastatic-free interval was defined as the time between date of primary breast cancer diagnosis and the date of first diagnosis of metastatic disease. Time to death was defined as the time between the date of questionnaire completion and the date of death. Two patients alive at last follow-up were excluded from the time to death analysis.

Descriptive statistics were used to examine the individual item scores of the EQ-5D-3L questionnaire. The health-state utility scores were calculated using the item scores and the valuation function developed by Dolan et al. based on the time trade-off method [31]. Health-state utility scores could range from -0.594 to 1.000, with negative scores implying health-states considered worse than death [32, 33]. The minimum clinically important change or difference (MID) on the EQ-5D utility score was set at 0.03, which is a widely used cutoff point [34].

Subgroups were compared using parametric (Student’s t-tests) and non-parametric (Mann–Whitney–Wilcoxon test) methods, respectively, for comparing two subgroups and using one-way analysis of variance (ANOVA) and Kruskal–Wallis test, respectively, for comparisons of more than two groups.

For assessing correlations between utility scores and time of metastatic disease and time to death, the Pearson test was used to calculate a correlation coefficient (*r*). The effect size of correlations was defined as weak (*r* = 0.20–0.39), moderate (*r* = 0.40–0.59), or (very) strong (*r* ≥ 0.60–1.0) [35]. For all statistical tests, an alpha of 0.05 was assumed for statistical significance.

## Results

### Patient, disease, and treatment characteristics

A total of 92 patients completed the EQ-5D-3L questionnaire between October 2010 and May 2011. Most patients were younger than 65 years (65%) and had no comorbidity (75%) (Table 1). The median metastatic-free interval was 19.9 months (95% confidence interval (CI) 13.1–26.8). Half of the patients (48%) received prior (neo)adjuvant systemic therapy, and 93% of the patients were actively being treated. Most patients (73%) had more than one metastatic site, in the majority of cases with visceral involvement. The median time to death counting from the date of questionnaire completion was 16.5 months (95%CI 10.2–22.8). At last follow-up, 2 patients were still alive and 90 had deceased.

**Table 1:** Patient, disease, and treatment characteristics (total *n* = 92)

Characteristic	No. of patients (%)
Age (years)	
< 65	60 (65%)
≥ 65	32 (35%)
Comorbidity	
No	69 (75%)
Any	23 (25%)
Cardiovascular	11 (12%)
Diabetes	4 (4%)
Lung disease	5 (5%)
Cerebrovascular	8 (9%)
Mobility	10 (11%)
Hormone receptor status	
Positive	7784%)
Negative	15 (16%)
HER 2-status	
Positive	21 (23%)
Negative	71 (77%)
Tumor characteristics	
HR+/HER2−	65 (71%)
HR+/HER2+	12 (13%)
HR−/HER2+	9 (10%)
HR−/HER2−	6 (7%)
Prior (neo-)adjuvant systemic therapy	
None	48 (2%)
Endocrine therapy (with or without targeted therapy)	24 (26%)
Chemotherapy (with or without targeted therapy)	37 (40%)
Metastatic-free interval	
De novo metastatic disease	14 (15%)
< 24 months	10 (11%)
≥ 24 months	68 (74%)
Total number of metastatic sites	
1	25 (27%)
≥ 2	67 (73%)
Metastatic sites^a^ at time of completion of the questionnaire	
Bone only	11 (12%)
Soft tissue without visceral or CNS involvement	17 (18%)
Visceral without CNS involvement	60 (65%)
CNS	4 (4%)
Current treatment	
None	6 (7%)
Endocrine therapy (with or without targeted therapy)	47 (51%)
Chemotherapy (with or without targeted therapy)	34 (37%)
Targeted therapy alone	5 (5%)
Number of palliative systemic treatments	
1	37 (40%)
2	20 (22%)
3	11 (12%)
≥ 4	24 (26%)
Number of palliative endocrine treatment lines	
1	49 (53%)
2	21 (23%)
3	9 (10%)
≥ 4	13 (14%)
Number of palliative chemotherapy lines	
1	62 (67%)
2	11 (12%)
3	11 (12%)
≥ 4	8 (9%)
Number of treatments (all, including neo/adjuvant therapy)	
1	25 (27%)
2	17 (18%)
3	14 (15%)
≥ 4	36 (39%)

^a^Sites of disease were classified in a mutually exclusive manner. Soft tissue localizations consisted of lymph nodes, skin and eye metastases. Visceral localizations consisted of liver, lung, pleura, peritoneum, gastrointestinal track, kidney and ovaries. Central Nervous System (CNS) localizations consisted of brain and leptomeningeal metastases

*CNS* Central Nervous System, *No* number, *HER2* Human Epidermal growth factor 2-receptor, *HR* hormone receptor, respectively, Estrogen or Progestogen-receptor

### Individual item scores

For the dimensions mobility, usual activities, and pain/discomfort, most patients reported some problems (level 2), while for the dimensions self-care and anxiety/depression most patients reported no problems (level 1). For all dimensions, extreme problems (level 3) were scarcely reported, occurring most frequently in the dimensions usual activity (15%) and pain/discomfort (10%) (Table 2).

**Table 2:** Individual item scores of the EQ-5D questionnaire in a cross-sectional study of patients with advanced breast cancer by age

	No problems/level 1	Some problems/level 2	Extreme problems/level 3	Total of some and/or extreme problems^a^
	*N* (%)	*N* (%)	*N* (%)	*N* (%)
Mobility				
All ages	43 (47%)	47 (51%)	2 (2%)	49 (53%)
< 65 years	34 (57%)	25 (41%)	1 (2%)	26 (43%)
≥ 65 years	9 28%)	22 (69%)	1 (3%)	23 (72%)
Self-care				
All ages	70 (76%)	20 (22%)	2 (2%)	22 (24%)
< 65 years	50 (83%)	9 (15%)	1 (2%)	10 (17%)
≥ 65 years	20 (63%)	11 (34%)	1 (3%)	12 (38%)
Usual activities				
All ages	22 (24%)	55 (60%)	14 (15%)	69 (75%)
< 65 years	16 (27%)	38 (64%)	5 (9%)	43 (73%)
≥ 65 years	6 (19%)	17 (53%)	9 (28%)	26 (81%)
Pain/discomfort				
All ages	30 (33%)	53 (58%)	9 (10%)	62 (67%)
< 65 years	21 (35%)	36 (60%)	3 (5%)	39 (65%)
≥ 65 years	9 (28%)	17 (53%)	6 (19%)	23 (72%)
Anxiety/depression				
All ages	48 (52%)	38 (41%)	6 (7%)	44 (48%)
< 65 years	36 (60%)	19 (32%)	5 (8%)	24 (40%)
≥ 65 years	12 (38%)	19 (59%)	1 (3%)	20 (63%)

^a^Some or extreme problem was defined as a level 2 or level 3 response on the EQ-5D questionnaire

*n* number of patients

On all dimensions, but most prominent in mobility (72% vs. 43%), self-care (38% vs. 17%), and anxiety/depression (63% vs. 40%), patients aged ≥ 65 years reported level 2 or 3 problems more frequently compared to younger patients (Table 2). Specifically, extreme problems (level 3) regarding the usual activities and pain/discomfort dimensions were reported by, respectively, 28% and 19% of patients aged ≥ 65 years, compared to 9% and 5% in the younger patient group. Overall, less than 10% of the younger patients reported severe problems on any of the dimensions.

### Utility scores

The utility scores for the whole cohort were not normally distributed but skewed to the right, with a mean of 0.602 (SD 0.312) and a median of 0.691 (IQR 0.244) (data not further shown). Therefore, we report results accounting for this non-normal distribution*,* i.e., using the described non-parametric models. Notably, parametric models produced consistent results (Table 3).

**Table 3:** Utility scores by patient, disease, and treatment subgroups

	No. of patients (%)	Mean	SD	Median	IQR	*p* value	*p* value
						Para-metric	Non-parametric
All	92 (100%)	0.602	0.312	0.691	0.244		
Age							
< 65 year	60 (65%)	0.660	0.271	0.743	0.194	0.026	0.017
≥ 65 year	32 (35%)	0.494	0.359	0.638	0.463		
Comorbidity							
No	69 (75%)	0.661	0.259	0.725	0.194	0.012	0.005
Yes	23 (25%)	0.426	0.392	0.620	0.561		
Tumor characteristics							
HR+ HER2−	65 (71%)	0.579	0.329	0.691	0.429	0.499	0.460
HR+ HER2+	12 (13%)	0.729	0.164	0.744	0.172		
HR− HER2+	9 (10%)	0.616	0.383	0.691	0.071		
HR− HER2−	6 (7%)	0.574	0.227	0.575	0.239		
Current treatment							
No systematic treatment	6 (7%)	0.734	0.137	0.743	0.107	0.245	0.196
Endocrine therapy (w/ or w/o targeted therapy)	47 (51%)	0.601	0.302	0.691	0.209		
Chemotherapy							
(w/ or w/o targeted therapy)	34 (37%)	0.550	0.354	0.661	0.344		
Targeted therapy only	5 (5%)	0.807	0.113	0.848	0.054		
Number of treatment lines (all)							
1	37 (40%)	0.586	0.371	0.691	0.332	0.439	0.675
2	20 (22%)	0.636	0.270	0.760	0.206		
3	11 (12%)	0.721	0.131	0.691	0.210		
≥ 4	24 (26%)	0.544	0.303	0.630	0.343		
Number of metastatic sites							
1	25 (27%)	0.629	0.333	0.689	0.192	0.629	0.722
≥ 2	67 (73%)	0.592	0.307	0.691	0.363		
Type of metastatic sites							
Bone only	11 (12%)	0.594	0.273	0.623	0.096	0.763	0.737
Soft tissue w/o visceral or CNS involvement	17 (18%)	0.532	0.450	0.691	0.209		
Visceral w/o CNS involvement	60	(65%)	0.620	0.272	0.691	0.296	
Central Nervous System (CNS)	4 (4%)	0.655	0.365	0.787	0.342		

*No.* number, *SD* standard deviation*, IQR* interquartile range, *HER2* Human Epidermal growth factor 2-receptor, *HR* hormone receptor, respectively, Estrogen or Progestogen-receptor, *w/* with, *w/o* without

There was a statistically significant difference between scores of patients aged < and ≥ 65 years (medians 0.743 versus 0.638; *p* = 0.017) (Table 3). Moreover, median utility scores were significantly worse for patients with versus those without comorbidity (yes versus no; 0.620 versus 0.725, *p* = 0.005). No statistically significant differences were observed between other subgroups.

**Fig. 1:**
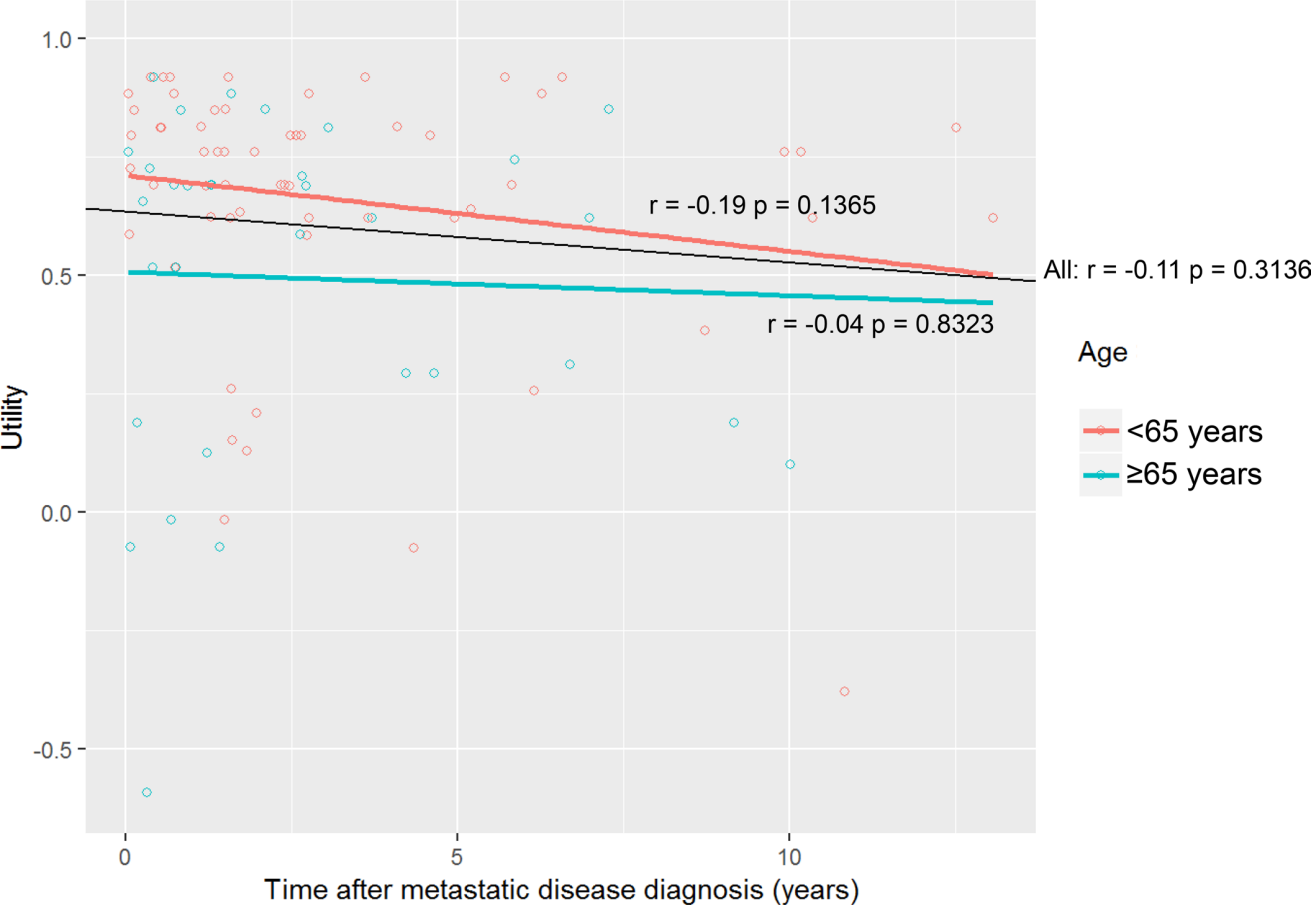
Utility versus time after metastatic disease diagnosis for the whole cohort (black line, *r* = -0.11 *p* = 0.3136) and by age subgroups < 65 years (red line, *r* = 0.19 *p* = 0.1365) versus ≥ 65 years (blue line, *r* = 0.04 *p* = 0.8323). (Color figure online)

**Fig. 2:**
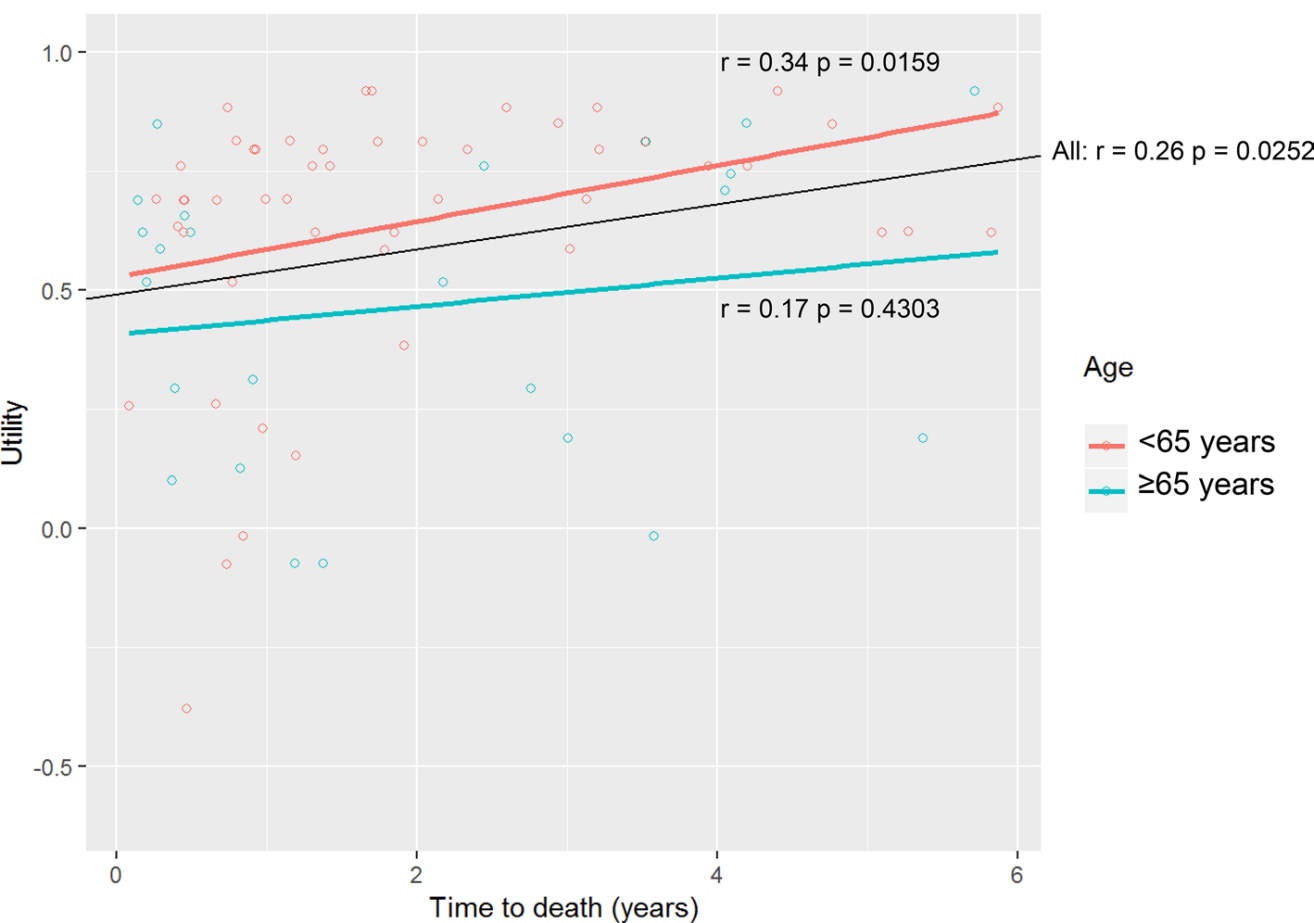
Utility versus time to death*, for the whole cohort (black line, *r* = 0.26 *p* = 0.0252) and by age subgroups < 65 years (red line, *r* = 0.34 *p* = 0.0159) versus ≥ 65 years (blue line, *r* = 0.17 *p* = 0.4304). *Only patients who deceased during the observation period were included. Two patients were still alive at last follow-up and were excluded from these analyses. (Color figure online)

No relation was identified between utility and time after metastatic disease diagnosis (Fig. 1; *r* = − 0.106, *p* = 0.3136) nor between utility and the number of prior lines of systemic therapy (data not shown). Interestingly, there was a positive relation between utility and the remaining survival time (Fig. 2; *r*: 0.260, *p* = 0.0252), especially in the subgroup of patients of patients < 65 years (*r*: 0.340, *p* = 0.0169).

## Discussion

This cross-sectional study evaluated the QoL in real-life of 92 ABC patients on different treatments (including no treatment). The median EQ-5D utility score was 0.691 (IQR 0.244), and differed significantly between subgroups based on age and the presence of comorbidity to both a statistically significant (*p* < 0.05) as well as a clinically relevant (difference in utilities > 0.03) extend, to the detriment of patients aged ≥ 65 years and patients with any comorbidity. Interestingly, we noticed a weak but significant positive correlation between the observed QoL and a patients’ remaining survival time (*r* = 0.260, *p* = 0.0252). Patients with a few months to live reported lower QoL as compared to those with a longer time to live, and this association was most clearly observed in the patients aged < 65 years (*r* = 0.340, *p* = 0.0169).

In clinical practice, it is difficult to determine the prognosis of an individual patient accurately. The decision on whether to start a new treatment line in case of progression is based on the doctor’s experience and perception and the patient’s preferences. The doctor’s perception of the patient’s wellbeing is reflected by the performance status ascribed to the general functioning of the patient. Indeed, performance status has shown to be related to treatment duration [36, 37] and overall survival [37–41] of patients with ABC within real-world studies. The observation that QoL can also be of assistance in guiding these decisions is interesting and should be further investigated in prospective studies.

Previous real-world studies among breast cancer patients using the EQ-5D report similar to slightly higher utility scores (median ranging from 0.64–0.82) [19, 20, 22, 24, 26, 42, 43] compared to our median (0.69). These differences in utility scores can partly be explained by the fact that most of these studies [22, 24, 26, 43] only included patients that were actively treated for their disease, leaving out the more vulnerable patients receiving supportive care. Additionally, the majority of these real-world studies [19, 20, 24, 42] also included patients with early breast cancer for which it can be expected that the QoL will be better due to less disease-related symptom burden. Furthermore, the mean age of patients within these trials varied, where studies with a lower mean age generally reported better utility scores; e.g., the study by Kim et al. reported a mean utility of 0.82 in a population with a median age of 49.3 years [43].

Factors found to have a significant association with QoL within previous observational studies for patients with breast cancer of various stages, including ABC, were age, [19, 20] fatigue, [19, 25, 26] financial difficulties, [19] pain, [19, 22, 25] body image, [25] comorbidities, [20],performance status, [23] type of therapy, [19–24] number of lines of therapy, [21, 24] location of metastases, [21, 22, 25], and disease progression [23, 26–28]. However, these associations were inconsistent, possibly due to differences in questionnaires used (EQ-5D, EORTC QLQ-C30 or -BR23, Patient Care Monitor, FACT-B), population’s health-related preferences, cultural dissimilarities, and methodology used in the valuation process [44]. Additionally, not all studies investigated the same variables, and within the investigated variables, impact varied between studies. This inconsistency in significance of the relation between the mentioned factors and the experienced QoL might be due to the lack of standardized methods, in combination with the observational nature of the studies.

Here, we found no association between the reported QoL and duration of metastatic disease and the number of prior lines of systemic therapy (data not shown). This stability of QoL with increasing number of treatment lines could be due to a correct selection of suitable patients for further therapy by their oncologists. Mostly, patients who are fit enough will be treated with further lines of treatment. Alternatively, the gradually increased therapeutic possibilities, the increased duration and amount of response to treatment resulting in better symptom control, could be an explanation of the lack of association between QoL and number of treatment lines.

The decision-making process on whether or not to continue treatment is also influenced by culture. Studies on EQ-5D value sets indicated that population-specific beliefs about health can contribute to differences in valuing a specific dimension of the EQ-5D as more or less important [44, 45]. Within a simulation study, Dutch respondents ascribed less weight than UK respondents to most dimensions of the EQ-5D, with the exception of the anxiety/depression dimension [45]. Another study comparing different value sets for the EQ-5D also found the Dutch value set was the only one out of 14 sets ascribing a worse health-state to a depressed patient compared to a patient with pain [44]. Several other studies among healthy as well as specific disease populations indicated that using different country-specific value sets produces substantially different results, and that despite a high level of correlation these tariffs cannot be used interchangeably [46–54]. More research is needed regarding the international transferability of utilities to ensure the economic evaluations underlying decision making are reliable and applicable to the intended population.

Thus, due to the large variation in methods used to assess QoL in breast cancer patients, [8, 14–16] there is a need for a more standardized approach. Therefore, the European guideline on the treatment of ABC urges the development of specific tools to evaluate QoL in ABC patients with attention to solid methodology [55]. The goal is to find a patient-centered way to measure QoL which incorporates the most relevant factors for patients, physicians, and decision makers with regard to drug approval and reimbursement. An important step forward is the central registration of patient-reported outcome data. For this purpose the EORTC Quality of Life Group developed a dedicated registry (PROMOTION) to identify, track, and analyze information about patient-reported outcomes (PRO), including QoL, of cancer patients included in randomized clinical trials since 2004 [56]. This database contains information regarding the assessment methodology, statistical design, and reported clinical and PRO results.

Our study is a cross-sectional study, and as a result changes in QoL during the disease course could not be investigated. Unfortunately, multivariate regression models were not performed due to the limited sample size of some subgroups and the skewness of the data. We intentionally mainly used descriptive statistics and (non)parametric tests, as regression models would be of limited usefulness in this case. Using a larger sample size and adopting multivariate regression models might be an informative step for future research to investigate a cause-effect relationship more profoundly. Furthermore, we only included patients that visited our outpatient clinic which could have led to an overestimation of the QoL scores. Finally, we did not have data on performance score and other possible relevant factors, such as toxicity. While highlighting the potential value of QoL in a similar role to performance status, such as in guiding patient decision making, correlation between the EQ-5D values and performance status assessments would have been worth mentioning. If highly correlated, using the EQ-5D could provide the advantage of covering more dimensions of QoL over the assessment of performance status. Conversely, performance score assessment is routinely done and might provide a quick and easy tool to guide decision-making. Strong points of our study are on the use of the preferred and validated generic instrument (EQ-5D), and that fact that our study is of important relevance for the ABC field, especially since it contains health-state utility data from routine clinical practice, which may be considered more representative data for guidance of decision-making than data from clinical trials.

In conclusion, within this real-world cross-sectional study, QoL was significantly associated with age, comorbidity, and remaining survival duration. The observation of a lower QoL in ABC patients, possibly indicating the last period of life, may assist clinical decision-making on timing of cessation of systemic antitumor therapy.

## Data Availability

Additional information on the Southeast Netherlands Advanced BREast cancer (SONABRE) Registry can be found at the website of the U.S. National Library of Medicine, ClinicalTrials.gov, using identifier number NCT03577197.
